# Phase I/II Trial of Ruxolitinib in Combination with Trastuzumab in Metastatic HER2 Positive Breast Cancer

**DOI:** 10.1007/s10549-021-06306-4

**Published:** 2021-06-24

**Authors:** Matthew Kearney, Lauren Franks, Shing Lee, Amy Tiersten, Della F Makower, Tessa Cigler, Prabhjot Mundi, Dow-Chung Chi, Anupama Goel, Pam Klein, Eleni Andreopoulou, Joseph Sparano, Meghna Trivedi, Melissa Accordino, Andrea Califano, Dawn L Hershman, Jose Silva, Kevin Kalinsky

**Affiliations:** 1 Columbia University Irving Medical Center, 630 W 168^th^ St, New York, NY 10032, USA; 2 Mailman School of Public Health, Columbia University, 722 W 168^th^ St, New York, NY 10032, USA; 3 Herbert Irving Comprehensive Cancer Center, Columbia University, 1130 St Nicholas Ave, New York, NY 10032, USA; 4 Icahn School of Medicine at Mount Sinai, 1 Gustave L. Levy Pl, New York, NY 10029; 5 Montefiore Medical Center, 111 E 210th St, Bronx, NY 10467; 6 Weill Cornell Medical, 1300 York Avenue, New York, NY 10065; 7 Winship Cancer Institute at Emory University, 1365 Clifton Road, Suite B4112, Atlanta, GA 30322

**Keywords:** Breast Cancer, Ruxolitinib, JAK2, HER2 positive

## Abstract

**Purpose:**

Preclinical data demonstrate STAT3 as an important regular in HER2+ tumors, and disruption of the IL6-JAK2-STAT-S100A8/S100A9 signaling cascade reduces HER2+ cell viability. Ruxolitinib is an FDA approved inhibitor of JAK1 and JAK2. We performed a phase I/II trial investigating the safety and efficacy of the combination of trastuzumab and ruxolitinib in patients with trastuzumab-resistant metastatic HER2+ breast cancer.

**Methods:**

Patients with metastatic HER2+ breast cancer progressing on at least 2 lines of HER2-directed therapy were eligible. The phase I portion determined the tolerable dose of ruxolitinib in combination with trastuzumab. The primary objective of the phase II was to assess the progression free survival (PFS) of the combination of ruxolitinib plus trastuzumab compared to historical control.

**Results:**

Twenty-eight patients were enrolled, with a median number of prior therapies of 4.5. Ruxolitinib 25mg twice daily was the recommended phase II dose with no DLTs. Of 26 evaluable patients in phase II, the median PFS was 8.3 weeks (95% CI: 7.1, 13.9). Among the 14 patients with measurable disease, 1 patient had a partial response and 4 patients had stable disease. Most of the adverse events were hematologic.

**Conclusion:**

While well-tolerated with a strong preclinical rationale, the combination of ruxolitinib and trastuzumab did not lead to an improvement in PFS compared to historical control in patients with trastuzumab-resistant metastatic HER2+ breast cancer.

## Introduction

HER2 is a transmembrane receptor tyrosine kinase which plays an important role in cellular proliferation, differentiation and survival [1]. There have been significant advances in the treatment of HER2 positive (HER2+) metastatic breast cancer. Trastuzumab is a recombinant monoclonal antibody targeting the extracellular domain of HER2, which reduces cellular proliferation via inhibition of several downstream signaling pathways [2] and enhances apoptosis through antibody dependent cellular cytotoxicity [3]. Since the approval of trastuzumab, a variety of agents currently have FDA approval for the treatment of HER2+ metastatic breast cancer, including pertuzumab, lapatinib, neratinib, ado-trastuzumab emtansine, fam-trastuzumab deruxtecan, tucatinib, and margetuximab. Despite these therapeutic improvements, most patients with metastatic HER2+ disease have tumors that ultimately progress on currently approved HER2 directed agents. As such, identifying treatment strategies with novel agents complementary to HER2 directed therapy would present an important non-chemotherapy option to patients whose tumors progress on standard therapy.

Using an integrative analysis of RNAi screens in combination with computational algorithms, preclinical data have identified that STAT3 is an important regulator of HER2+ tumors [4]. Further analysis has demonstrated that activation of HER2 results in high levels of IL-6 production, which subsequently generates an autocrine loop that leads to the activation of the JAK/STAT pathway and STAT3 induction. Additional studies have reinforced that STAT3 activation plays a role in breast tumorigenesis [5–7]. S100A8 and S100A9 are direct targets of STAT3, playing a critical role in HER2-mediated oncogenesis through promotion of proliferative and survival signal transduction pathways. As disruption of the IL6-JAK2-STAT-S100A8/S100A9 cascade impacts HER2+ cell viability, these data serve as the rationale for targeting this pathway in patients with HER2+ breast cancer.

Ruxolitinib is a potent inhibitor of both JAK1 and JAK2 and is approved for the treatment of intermediate to high risk myelofibrosis [8], hydroxyurea-refractory polycythemia vera [9] and steroid-refractory acute graft versus host disease [10]. JAK1 and JAK2 are receptor Janus kinases that are members of the JAK/STAT signaling pathway. Activation of this pathway leads to the recruitment of several cytoplasmic signal transducers and activators of transcription to the nucleus, which subsequently modulates gene expression in proteins responsible for cell survival and proliferation [11]. Pre-clinical data have demonstrated that treatment of HER2+ breast cancer cell models with ruxolitinib decreases STAT3 phosphorylation, decreases tumor growth *in vivo* and is synergistic when combined with trastuzumab [4]. Thus, we initiated a phase I/II trial investigating the safety and efficacy of the combination of trastuzumab and ruxolitinib in patients with metastatic trastuzumab-resistant HER2+ breast cancer.

## Methods

### Patient Population & Eligibility Criteria

Entry criteria for this multi-center, investigator-initiated trial included women and men > 18 years of age with histologically or cytologically confirmed locally recurrent or metastatic HER2+ breast cancer, per ASCO-CAP guidelines [12]. Patients must have received a minimum of 2 lines of HER2-directed therapies in the metastatic setting including trastuzumab, pertuzumab, and ado-trastuzumab emtansine unless medically contraindicated or declined by patient or treating physician. Patients who relapsed within 12 months of completing pertuzumab or ado-trastuzumab emtansine in the operable setting were considered as having progressed on that regimen. Measurable or non-measurable disease was allowed. Additional eligibility criteria included: Eastern Cooperative Oncology Group performance status (ECOG PS) 0-2; left ventricular ejection fraction ≥ 50%; baseline corrected QT interval ≤ 480 milliseconds on EKG; no prior experience with JAK2, STAT3, or IL-6 antagonists; normal organ and marrow function; and a life expectancy of at least 12 weeks. Any systemic anti-cancer therapy or radiotherapy must have been completed at least 2 weeks prior to study treatment initiation. Asymptomatic patients with metastatic brain disease were eligible if they had been on a stable dose of corticosteroids for treatment of brain metastases for at least 14 days prior to study entry.

### Study Treatments

Ruxolitinib was administered orally twice daily (bid) every day for 3-week (21-day) cycles. Trastuzumab was administered intravenously on day 1 of each cycle. If the patient had not received trastuzumab within the past 28 days, the initial loading dosage was 8 mg/kg of trastuzumab. Otherwise, the patient started on an initial dose of 6 mg/kg, with all subsequent doses of trastuzumab at 6 mg/kg. Pre-medications to mitigate infusion reactions to trastuzumab were allowed and administered 30 minutes prior to administration of trastuzumab. There were no breaks between cycles. In the phase I adaptive design, four dose levels were selected to find an acceptable dose of ruxolitinib in combination with trastuzumab: 10 mg bid, 15 mg bid, 20 mg bid, and 25 mg bid. The initial dose level of ruxolitinib was 20 mg bid. Phase II used the acceptable dose level of ruxolitinib in combination with trastuzumab determined in phase I.

### Assessments

Patients were assessed with radiographic imaging every nine weeks (3 cycles) using Response Evaluation Criteria in Solid Tumors (RECIST) guidelines (version 1.1) [13]. Patients were classified as having: Complete Response (CR), Partial Response (PR), Stable Disease (SD), or Progressive Disease (PD). Patients had physical exams and were assessed for adverse events, vitals and performance status weekly in the first cycle of treatment and at the beginning of each cycle thereafter. Adverse events were graded using the NCI Common Terminology Criteria for Adverse Events, CTEP version 4.0. Patients in phase I were assessed for dose limiting toxicities (DLT) during the first cycle of treatment (21 days). A DLT is defined as any grade 3 non-hematologic toxicities or any grade 4 hematologic toxicities directly related to the combination.

### Study Design

This study was a non-randomized, open label phase I/II trial designed to evaluate the safety and efficacy of the combination of ruxolitinib plus trastuzumab. Patients were enrolled from four centers in New York: Columbia University Irving Medical Center, Weill Cornell Medical Center, Montefiore Medical Center, and Mt. Sinai Medical Center. This study was conducted with approval from the Institutional Review Board at each enrolling center and is registered under NCT02066532 at ClinicalTrials.gov.

#### Phase I:

The phase I study objective was to determine an acceptable dose of ruxolitinib in combination with trastuzumab. The maximum tolerated dose (MTD) combination was defined as the dose combination of ruxolitinib and trastuzumab associated with a target probability of dose limiting toxicity (DLT) of 25%. Time to Event Continual Reassessment Method (TITE-CRM) was used to assign doses and estimate the MTD using an empirical dose-toxicity model. The design parameters were calibrated to select a dose that yields between 16% and 34% DLT.

#### Phase II:

The primary objective of the phase II was to estimate the progression free survival (PFS) of the combination of ruxolitinib plus trastuzumab. PFS was defined as time from patient registration until disease progression or death from any cause. Key secondary endpoints included overall response rate (ORR), clinical benefit rate (CBR), and safety. CBR was defined as CR, PR, or SD for > 24 weeks. Phase II included any phase I patient who was treated at the recommended phase II dose level (RP2D).

### Statistical Analysis and Sample Size Determination

All patients are considered evaluable for toxicity from the time of their first treatment with ruxolitinib and trastuzumab, and all patients regardless of therapy initiation or study compliance were considered evaluable for the phase II efficacy analysis. For the phase I study, a sample size of 10 patients yielded correct dose selection probability of at least 50% in the most likely scenarios which assumed dose level 4 or 5 had a 25% probability of DLT. For the phase II study, the sample size was estimated using the primary outcome of PFS and assuming an exponential survival. Assuming a historical PFS control of 8 weeks with single-agent HER2-targeted therapy in metastatic HER2+ breast cancer after progressing on trastuzumab-based therapy [14] and that patients receiving the combination of ruxolitinib at the MTD dose plus trastuzumab will have a PFS of at least 13 weeks, with a one-sided alpha of 0.05 the study had 80% power to detect a significant difference with 30 patients enrolled.

PFS was estimated using the Kaplan-Meier method and reported with 95% confidence intervals. Patients who discontinued prior to their first scan were censored at their last follow up and those who had longer follow up were censored at the time of the last progression free scan. ORR was evaluated by tabulating the number of evaluable patients with CR and PR out of all patients evaluable for efficacy and exact binomial 2-sided 95% confidence intervals. CBR was estimated using proportions achieving CR, PR or SD >24 weeks with exact 95% confidence interval. Continuous descriptive statistics were summarized using minimum, median, and maximum values, and categorical variables were summarized using frequencies and percentages. Adverse events were tabulated and analyzed considering the number of patients experiencing the adverse event and the highest grade experienced by each patient.

## Results

### Phase I/II

**Figure 1.**
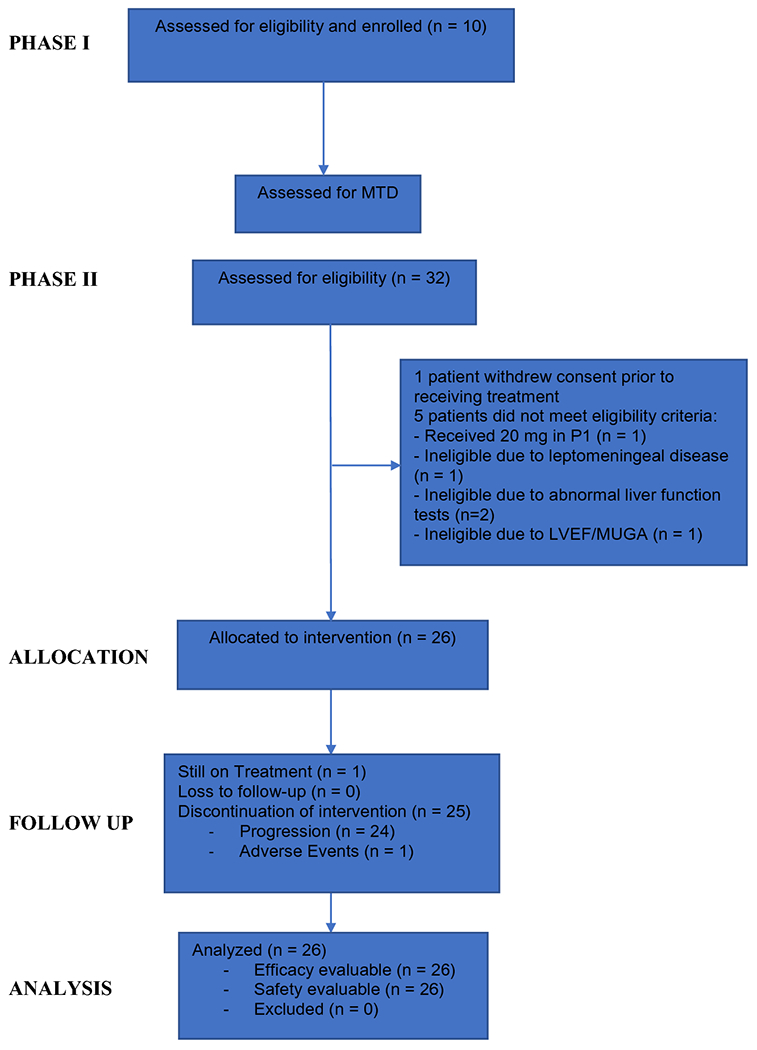
Patient CONSORT Diagram

From October 2014 to August 2018, we assessed 32 patients for eligibility in this phase I/II trial, of whom 28 patients met eligibility criteria. The dataset was locked on May 20, 2020. During phase I, 10 patients were enrolled, and MTD was estimated. The first patient was treated at the starting ruxolitinib dose level: 20 mg bid. In the absence of DLT, the dose was escalated and the remaining nine patients were assigned to 25 mg bid. By the completion of phase I, the MTD was not reached, no DLTs were observed, and 25 mg bid was selected as the RP2D for ruxolitinib. Phase II was terminated early due to slow accrual. In total, 27 patients were enrolled and one patient withdrew consent prior to treatment, resulting in 26 patients evaluable for phase II, including the 9 patients from phase I treated at the RP2D of 25 mg bid. The CONSORT diagram is demonstrated in Figure 1.

In phase II, the median age was 56 (32-77), 24 patients (92%) were postmenopausal, 19 patients (73%) had hormone receptor-positive (HR+)/HER2+ breast cancer, and 14 (54%) had an ECOG PS of 0. These baseline characteristics are summarized in Table 1. Eligible patients received between 1 and 10 lines of prior therapy in the metastatic setting, with a median of 4.5 lines. Twenty-three patients (88%) received prior pertuzumab and 25 patients (96%) previously received ado-trastuzumab emtansine.

### Safety

Table 2 lists the maximum grade of adverse events identified as definitely, probably, or possibly related to the study drug in at least 10% of the 26 phase II patients. There was no grade IV or higher study drug related adverse event reported. Anemia was the most frequently observed adverse event. Thirteen patients (50%) experienced anemia, of whom, 7 (27%) had a grade III toxicity. Other commonly experienced adverse events included neutropenia (38%), fatigue (35%), white blood cell count decrease (35%), thrombocytopenia (31%), AST increase (15%), and diarrhea (15%). All grade III events were hematologic toxicities, except for 1 patient with an increase in aspartate aminotransferase. One patient had grade II heart failure, with an ejection fraction decrease by 17%, and therefore permanently discontinued trastuzumab after two cycles. Notably, the patient remained on ruxolitinib 20 mg BID as a single agent with SD for a total of 30 cycles. In total, 7 patients (27%) had dose reductions, 13 patients (50%) required dose holds, and 21 patients (81%) were at least 80% compliant.

### Efficacy

**Figure 2.**
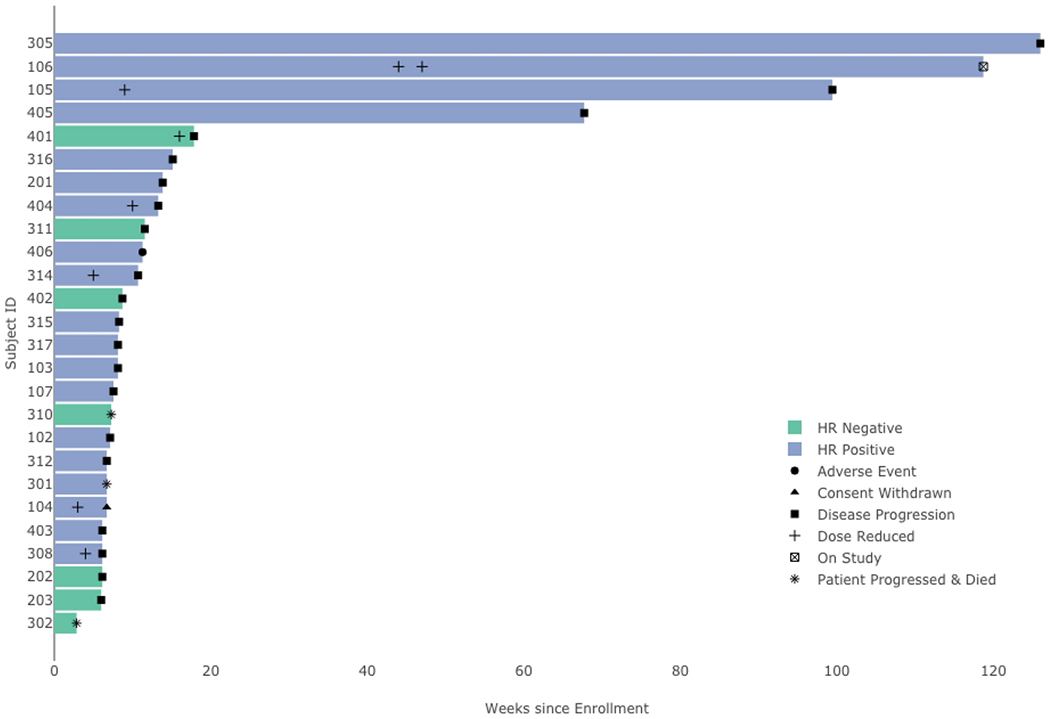
Swimmer’s Plot for the 26 evaluable patients in Phase II

During the phase II portion of the study (n = 26 patients), one patient was removed from the study drug due to adverse events related to a temporal cranial resection wound infection after 11.2 weeks and was no longer thought to be a suitable study candidate. One patient, who died after 2.8 weeks, developed legionella pneumonia after starting the trial; however, the patient’s death is attributed to disease progression and not attributable to study medication. 24 patients discontinued treatment for progression and one patient was removed from treatment due to adverse events. One patient remains on study treatment. The swimmer’s plot depicted in Figure 2 describes each patients duration on treatment and all relevant events on trial. The median PFS was 8.3 weeks (95% CI: 7.1, 13.9). Out of the 26 patients, 1 patient with estrogen receptor-positive (> 95%), progesterone receptor-positive (60%)/HER2 amplified breast cancer achieved a PR (ORR 4%). This patient’s tumor had progressed on 7 previous lines of therapy. Four of the 26 evaluable patients (15.4%, 95% CI: 4%, 35%) experienced SD or better for greater than 24 weeks, of whom two had non-measurable disease. These four patients had HR+/HER2+ breast cancer and remained progression free for 67 weeks or more, with one patient still on study after 131 weeks on treatment as of May 2020.

**Figure 3:**
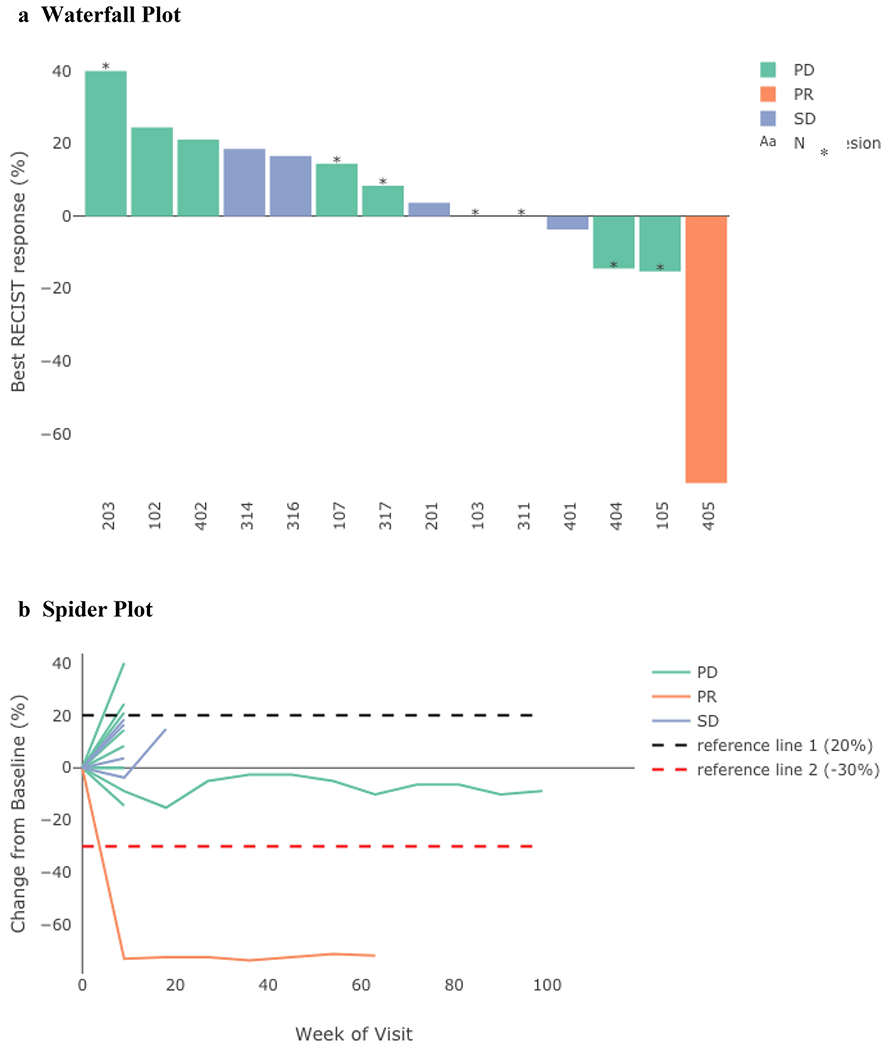
Response to ruxolitinib plus trastuzumab in the 14 patients with measurable disease and at least one scan post-baseline

Among the 26 patients, 2 patients had non-measurable disease at baseline, of whom one progressed and one remained on trial. Among the remaining 24 patients with measurable disease, 10 discontinued treatment before their first scan past baseline. Each patient’s best RECIST response is described in Figure 3a, categorized by the patient’s RECIST status. Figure 3b illustrates the change in tumor size relative to baseline over the duration of the trial in these 14 patients, categorized by RECIST status.

## Discussion

In this phase I/II trial, the addition of ruxolitinib to trastuzumab in patients with HER2+ metastatic tumors that previously progressed did not meet its primary endpoint of improvement in PFS as compared to historical control with a median PFS of 8.3 weeks (95% CI: 7.1, 13.9). While the combination of ruxolitinib and trastuzumab was well tolerated with no unexpected adverse events, the study was terminated early due to slow accrual. One patient had a partial response to the combination of ruxolitinib and trastuzumab and 4 patients had a best response of stable disease among those with measurable disease.

It has been demonstrated through a combination of RNAi screens and network analysis that the IL-6-JAK-STAT3-S100A8/9 signaling pathway contributes to HER2+ breast tumorigenesis in preclinical models [4]. HER2 overexpression in transformed ErbB2/MCF-10A cell lines was associated with increased phosphorylation in various members of the STAT family, including STAT3. STAT3 inhibition was associated with reduced viability of HER2 mutated cells in mouse models and treatment with ruxolitinib was associated with reductions in STAT3 phosphorylation and induction of apoptosis. Despite strong pre-clinical rationale, translation to clinical benefit was not seen with this combination in this study population. It is possible that an inability to effectively inhibit HER2 signaling with trastuzumab in heavily pretreated patients limited our ability to see a signal of benefit. Ruxolitinib in combination with novel HER2 agents, which are more effective at inhibiting HER2 signaling in pre-treated patients, could be a future area of investigation.

The majority of patients on this trial had HR+/HER2+ breast cancer. Pre-clinical data have demonstrated higher levels of baseline STAT3 phosphorylation in MCF-7 cell lines that are resistant to tamoxifen compared to control MCF-7 cell lines, suggesting contribution of the JAK-STAT pathway in the development of tamoxifen resistance and in HR+ models [15]. In this study, the addition of ruxolitinib to both tamoxifen-resistant and control MCF-7 cells inhibited STAT3 activation in a concentration dependent manner. Ruxolitinib additionally inhibited cellular proliferation and tumor growth in the tamoxifen-resistant line but not the control lines. A similar study using a HR+/HER2+ model demonstrated concentration-dependent inhibition of cell growth with ruxolitinib that was synergistic when combined with calcitriol [16]. Lastly, tocilizumab, an IL-6 antagonist, has been evaluated in pre-clinical studies using HR+ models [17]. Tocilizumab reversed tamoxifen resistance in MCF-7 and ZR-75 cell lines with a splice variant known to induce tamoxifen resistance and increased IL-6 and STAT3 signaling, which was redemonstrated in an animal model.

This is the first trial to evaluate ruxolitinib in patients with HER2+ metastatic breast cancer. Ruxolitinib has been evaluated as a single agent in metastatic triple negative breast cancer (TNBC) [18]. Ruxolitinib at a dose of 25mg bid was evaluated in patients with metastatic TNBC selected by STAT3 phosphorylation positivity by immunohistochemistry. There were no responders in the 21 patients enrolled and the median PFS was 1.2 months despite a reduction in phosphorylated STAT positive cells, suggesting on-target activity. It was hypothesized that both intra-tumoral heterogeneity and incomplete inhibition contributed to lack of responses. A randomized phase II trial investigated the combination of ruxolitinib or placebo with capecitabine in patients with metastatic HER2 negative breast cancer and elevated markers of systemic inflammation [19]. Median OS and PFS were not improved with the combination, though exploratory analysis suggested an improved ORR in the ruxolitinib group and healthcare related quality of life was better in the ruxolitinib group. The combination of ruxolitinib with weekly paclitaxel is currently being investigated in a phase I/II trial in patients with metastatic inflammatory TNBC [20]. Given STAT3 is a target of IL-6 and the well described association of poorer outcomes in patients with solid tumors and higher levels of systemic inflammatory scores [21], ruxolitinib has been evaluated in several solid tumor types outside of breast cancer which have all not demonstrated a significant improvement in clinical outcomes [22–25].

The combination of ruxolitinib and trastuzumab was well tolerated. The most common adverse events were hematologic toxicity, with 27% of patients experiencing grade 3 anemia and 23% of patients experiencing grade 3 neutropenia. There were no grade 4 events and no patients required drug discontinuation secondary to ruxolitinib. Cytopenias are a well-documented effect of ruxolitinib [8, 9], which are typically managed with dose reductions and periodic interruptions of drug but rarely require drug discontinuation, as also demonstrated in this phase II trial with the high compliance rate.

The population treated on this trial were racially/ethnically diverse and heavily pre-treated, with a median number of prior therapies of 4.5. Patients with tumors that progressed on trastuzumab, pertuzumab and trastuzumab emtansine have poor outcomes, with median PFS of approximately 6 months and ORR 20-30% [26, 27] until the recent approval of trastuzumab deruxtecan [28] and tucatinib based combinations [29]. While the primary endpoint of this phase II trial was not met, four patients experienced prolonged stable disease and remained progression free for at least 67 weeks. One patient had a prolonged stability of disease with single agent ruxolitinib despite holding trastuzumab due to cardiomyopathy. One patient had a PR after 7 prior lines of therapy. Limitations to this study include limited pharmacokinetic sampling as well as pharmacodynamic evaluation, as there were limited matched pre-treatment and on- or post-treatment samples for biomarker assessment. Ideally, future biomarker analyses will identify a subgroup of patients who benefit from JAK2 inhibition.

In conclusion, while tolerable, the combination of ruxolitinib and trastuzumab did not improve PFS as compared to historical control in patients with trastuzumab resistant HER2+ metastatic breast cancer. Despite recent advances in patients with pre-treated HER2+ metastatic breast cancer, continued investigation of novel therapies in this patient population remains an unmet need.

## Figures and Tables

**Table 1.** Baseline Characteristics for the 26 evaluable patients in Phase II

Attribute	N (%)
Age, median (range), years	56 (32, 77)
Race	
Asian/Indian	2 (8)
Black	6 (23)
White	15 (58)
Unknown/Other	3 (11)
Ethnicity	
Hispanic	3 (11)
Non-Hispanic	21 (81)
Not Reported	1 (4)
Unknown	1 (4)
Hormone Receptor Status	
Positive	19 (73)
Negative	7 (27)
ECOG PS	
0	14 (54)
1	12 (46)
Metastatic at Time of Diagnosis	14 (54)
Post-menopausal	24 (92)
Prior Systemic Therapy	
Prior Metastatic Regimens, median (range)	4.5 (1, 10)
Prior pertuzumab	23 (88)
Prior ado-trastuzumab emtansine	25 (96)

Abbreviations: ECOG PS: Eastern Cooperative Oncology Group Performance Status

**Table 2.** Treatment-Related Adverse Events in ≥ 10% of Patients

Adverse Event	Grade 1	Grade 2	Grade 3	Total
*Anemia*	1 (3)	5 (19)	7 (27)	13 (50)
*Neutrophil count decreased*	-	4 (15)	6 (23)	10 (39)
*Fatigue*	6 (23)	3 (11)	-	9 (35)
*WBC decreased*	4 (15)	4 (15)	1 (4)	9 (35)
*Platelet count decreased*	5 (19)	2 (8)	1 (4)	8 (31)
*Aspartate aminotransferase increased*	2 (8)	1 (4)	1 (4)	4 (15)
*Diarrhea*	2 (8)	2 (8)	-	4 (15)
*Alanine aminotransferase increased*	1 (3)	1 (3)	1 (3)	3 (11)
*Bloating*	-	3 (11)	-	3 (11)
*Weight gain*	1 (3)	2 (8)		3 (11)
*Dizziness*	2 (8)	1 (3)		3 (11)
*Generalized muscle weakness*	2 (8)	1 (3)		3 (11)
*Blood and Lymphatic system disorders*	3 (11)			3 (11)
*Dyspnea*	2 (8)	1 (3)		3 (11)

Abbreviations: WBC: white blood cell

## Data Availability

The datasets generated and/or analyzed during the current study are available from the corresponding author on reasonable request.
